# Relative validity of a semi-quantitative food frequency questionnaire for Singaporean toddlers aged 15–36 months

**DOI:** 10.1186/s40795-018-0252-9

**Published:** 2018-12-11

**Authors:** Cameron Allan, Ummi Hani Abdul Kader, Jowynn Yu Ying Ang, Leilani Muhardi, Smita Nambiar

**Affiliations:** 1 School of Exercise & Nutrition Sciences, Queensland University of Technology, Victoria Park Road, Kelvin Grove, QLD 4059 Australia; 2 Early Life Nutrition, Danone Nutricia Research, 30 Biopolis St, Matrix Building #05-01B, Singapore, 138671 Singapore; 3 Healthcare Nutrition Science, Danone Nutricia Sari Husada, 15th Fl, Cyber 2 Building, Jl. HR Rasuna Said no. 13, Jakarta, Indonesia

**Keywords:** Food frequency questionnaire, Toddler, Validation, Singapore, Nutrient intake

## Abstract

**Background:**

There is presently no simple tool for use in large epidemiological studies to understand the food and nutrient intakes of Asian toddlers. This study aimed to assess the relative validity of a semi-quantitative food frequency questionnaire (sqFFQ) developed for multi-ethnic Singaporean toddlers aged 15–36 months.

**Methods:**

Ninety-one parents completed the sqFFQ and a 2-day weighed food record as the reference method. Intake of energy and 25 nutrients were determined for each method and compared using Pearson correlations corrected for attenuation, Bland-Altman plots, and weighted kappa according to quartiles; sqFFQ calibration was performed using multivariable linear regression.

**Results:**

Deattenuated correlations for energy and all nutrients were acceptable (r = ≥0.30, *p* < 0.001). The sqFFQ was highly reproducible, but significantly overestimated intake of energy and all nutrients except vitamin A. Bland-Altman plots showed wide limits of agreement for energy and all nutrients. Weighted kappa ranged from 0.12 (slight) to 0.53 (moderate). After calibration, deattenuated correlations improved for energy and 10/25 nutrients, with no change or a slight decline for the remainder, including one falling to *r* = 0.27. Limits of agreement narrowed for energy and all nutrients, and except for DHA, median intakes were not significantly different except for vitamin A, enabling population estimates of absolute intakes. Weighted kappa improved overall; energy and 16 nutrients now had moderate agreement (0.41–0.60), while 9 nutrients had fair agreement (0.21–0.40).

**Conclusions:**

The Singaporean toddler semi-quantitative food frequency questionnaire is suitable for ranking nutrient intakes of Singaporean toddlers in larger epidemiological studies. However, for population estimates of absolute nutrient intakes, it is recommended that a subsample within a cohort complete weighed food records for calibration purposes.

**Trial registration:**

This study was registered retrospectively on clinicaltrials.gov on 3rd May 2017 (identifier code: NCT03138330).

## Background

Toddlerhood is a critical period during the lifecycle. Defined here as children aged 12–36 months, this phase is marked by rapid growth, maturation of organs and increasing levels of physical activity [1]. Relative to their body size, toddlers have high nutritional requirements [1]. Any deficiencies or excesses in macro- and micronutrients that occur during this critical period can have lasting negative consequences later in life. Conditions such as iron deficiency and obesity are prevalent in developed and developing countries, and can often exist in parallel [2]. In addition to this, toddlers are establishing healthy eating habits as they transition from an infant diet to the family diet [3]. Therefore, insights into food and nutrient intakes of toddlers are extremely important. Dietary data collection can be integrated into clinical and epidemiological studies to understand the food and nutrient intake patterns of a population. Such information can help with the development of dietary guidelines, assess if children are meeting recommendations and if any diet-disease relationships exist.

Depending on study objectives, there are several different methods for collecting dietary information. These methods are similar in adults and children, however, with the exception of nutritional biomarkers, dietary information is obtained from a proxy (parent or guardian), especially if the child is under ten years of age [4]. The food record (FR) and FFQ are two examples of dietary assessment methods commonly used in epidemiological studies involving children [4].

The FR collects information current food intake and is used to estimate nutrient intakes [5]. Participants keep a diary of all foods and beverages consumed in a day, along with quantities that are estimated or weighed (WFR). Food records can be burdensome on participants due to the level of detail required and multiple days of recording. This can be especially challenging when toddlers are involved, as they may not eat the same foods as the rest of the family and different carers may be involved at various mealtimes, thus resulting in inconsistent reporting. For these reasons, the FR is one of the more tedious and expensive nutritional tools to implement and analyse [5, 6].

The FFQ differs significantly from the FR, as it retrospectively gathers information on habitual food intake. [7] The FFQ consists of a finite list of foods consumed by a particular population and participants indicate how often they consume these foods. Intake can also be crudely quantified [7]. The tool is inexpensive to administer; simple to complete and analyses is more straight forward. This makes it a useful tool in large population studies where the intention is to rank individuals according to their intakes and then seek associations between diet and disease [7]. Some limitations of the FFQ include: overestimation of nutrient intakes at the individual level; reliance on the user’s memory to recall past intake; its use is restricted to a specific population; it requires regular updating and it needs to be validated [5, 7].

There are a limited number of FFQs available for toddlers. Studies involving the development and validation of FFQs have been conducted in North America [8–15], Europe [16–23], Australia and New Zealand [24–26]. The age ranges included in these studies were 1.5–4 years. Even fewer FFQs are available for children in Asia [27, 28], of which none involve children less than three years of age. Therefore, presently, there is no simple tool for use in large epidemiological studies to understand the food and nutrient intakes of Asian toddlers.

To address this gap, a multi-ethnic sqFFQ for Singaporean toddlers was recently developed [29], but yet to be validated. The purpose of the present study was to validate this new tool for use among Singaporean toddlers aged 15–36 months. The most common reference method for the multi-nutrient validation of a sqFFQ designed for young children is the FR [30, 31]. The Singaporean toddler sqFFQ was assessed for its ability to rank and estimate nutrient intakes relative to the WFR for energy and 25 nutrients that are important for growth and development during this critical period.

## Methods

### Sampling, recruitment and participants

As studies involving the validation of nutritional tools among toddlers are limited in Asia, sample sizes used in other similar studies were used as a guide. Additionally, Cade and colleagues (2002), suggested at least 50–100 subjects are required for each demographic group, particularly if Bland Altman analyses and correlations are used; increasing the sample size beyond this would not strengthen correlations [31]. As the sqFFQ was designed as one questionnaire for a multiethnic sample (all races have access to many different types of foods and cuisines), a convenience sample of approximately 100 subjects and their primary caregiver was consecutively recruited over twelve months (December 2015 to November 2016). For inclusion into the study, toddlers had to be healthy, 15–36 months of age and of Chinese, Malay or Indian ethnicity (the predominant ethnic groups in Singapore) [32]. Children with any acute or chronic illnesses that affected food intake were excluded, as were children with one or both parents who did not meet the ethnicity criteria. This was to avoid over-representation of a minority group (3.2% of the population in 2016) [32], and also because the food list in the sqFFQ was based on food consumption of the three main races. Recruitment was from 15 months of age because the sqFFQ asks about habitual food intake over the last three months and only information from 12 months onwards was of interest.

Children were recruited via day-care centres. A convenience sample of 74 centres (35 government-based and 39 private) across the island were selected. Only 16 centres agreed to participate (reasons for non-participation included: too busy with administrative duties, committed to other studies, the principals felt they were not at liberty to authorise the study (headquarters had to be involved), or, simply not interested). These 16 centres provided approximately 260 children who met our inclusion criteria, of which, only 46 responded to the invitation letter (we are uncertain if invitation letters were distributed to all eligible children). To increase the speed of recruitment, the snowball technique was introduced, so current participants could refer others and research staff could ask colleagues and their friends to spread the word on the study. Sixty-six parents expressed interest via this method (*n* = 112).

Once the caregiver returned the participation form, the study research assistant arranged a face-to-face meeting to fully explain the purpose of the study, how to fill in a series of different questionnaires and obtain signed consent. Participants were given two weeks to complete the study components and received a $75 (Singapore dollars) shopping voucher for their participation.

Participants filled out a series of questionnaires in order to meet several study objectives. The questionnaires relevant to the objective of this study are described below.

### Initial questionnaire

The initial questionnaire was completed during the first face-to-face meeting. This questionnaire aimed to capture information on each parent’s weight, height, education level and combined household income. Parents self-reported their child’s birth weight and length and current weight and length/height. Parents could use the most recent child weight and height/length measure noted in the child’s health book (if it was in the last 2 weeks); however, they were encouraged to have the child measured at a local clinic during the study period. Study staff did not do the measurements because it required the child to be present at the initial meeting, which added another level of complexity to the recruitment. As anthropometric data were not crucial to the validation analyses and mainly collected to describe the population, self-reported data were deemed sufficient.

### Weighed food record

Parents were asked to record food intake for two non-consecutive days (one weekend, one weekday), as previously recommended [33–35]. Full instructions were given verbally during the initial meeting and detailed written instructions accompanied the WFR templates. Parents indicated the day, date, time of meals, meal occasion, description of all the foods offered, portion consumed, and place it was consumed. Extra pages were included for recipes and supplements used that day. Emphasis was placed on the level of detail required when describing the food types, recipes, cooking methods (including the addition of salt, seasonings, fats and oils) and brands. If the child was breastfed, mothers were asked to record the minutes the child latched on. Each participant was given digital kitchen scales which could register weights of 1 g to 5000 g (unnamed; model SF-2012) and they were showed how to tare the weight of plates/cups/bowls before weighing the food and weighing leftovers. In the instance where a meal was eaten away from the home, and the scales could not be used, parents were asked to describe portions in relation to standard cup and spoon measures, or, the standard bowl measure used in the sqFFQ (parents were shown what these were at the initial meeting). For meals consumed while the child attended day care, the research assistant obtained details from the facility, as meals are supplied by the facility. If another carer oversaw a mealtime, they were asked to fill out the details in the diary. All the food WFRs were reviewed in person or via phone call. At the end of the review, the parent was asked if the child’s intake was usual, more than usual or less than usual. If, after review, the record was still deemed as poor quality, then it was excluded from analyses.

### Semi-quantitative food frequency questionnaire

The sqFFQ was an original design, with food lists and portion sizes developed in a previous study [29]. Briefly, the sqFFQ food list was derived by interviewing 30 mothers (ten from each ethnic group mentioned above) in a focus group setting. The mothers were asked about the child’s habitual intake and were also asked to complete 3-day food diaries. Over 500 different foods, typical portion sizes and utensils were reported from the interviews and diaries. It was decided that one food list would be used for all three races. This was because Singapore is a multicultural society and all races could easily access any type of food and cuisine. The final sqFFQ consisted of 99 items, including single and composite items, as well as items where foods of a similar type and nutrient profile were grouped together (for example in the vegetables section, vegetables were grouped as bulbs, tubers, root, stem, fruit, seeds, with examples provided for each; certain items in other food groups were separated based on their fat, sugar and fibre content). These 99 items were then divided across 11 food groups: breads and cereals, vegetables, fruits, legumes and nuts, meat/poultry/fish and alternatives, dairy and alternatives, snacks, fast foods, beverages (other than dairy and alternatives), salty and sweet seasonings including fats and oils used in cooking, and supplements. Within each group, an open question was included where participants could add other foods to the list. Portion sizes commonly used for toddlers were listed next to each item. Frequency responses started at “Never” and increased across 10 categories to a maximum of “>6 times per day”. Verbal and detailed written instructions were given, including illustrations showing the portion sizes referred to in the food lists and dimensions of common utensils. An appendix was included with photographs and descriptions of approximately 50 different foods listed in the sqFFQ to further guide parents. Reproducibility was assessed by asking participants to complete a second sqFFQ. As there were no guidelines indicating whether the full sample was needed for reproducibility, or, a proportion of the sample was sufficient, 20 % of the sample were asked to complete a second sqFFQ one to two weeks later [27, 34, 36].

The completed questionnaires were reviewed, particularly if the portions, when totalled, exceeded what was recommended for this age group.

### Nutrient analysis

Energy and 25 nutrients were of interest in this study: protein, total fat, saturated fat (SFA), monounsaturated fat (MUFA), polyunsaturated fat (PUFA), docosahexaenoic acid (DHA), total carbohydrate (CHO), total sugars, fibre; vitamins: A, thiamine (B1), riboflavin (B2), niacin (B3), dietary folate equivalents (DFE), cobalamin (B12), C, E; minerals: calcium (Ca), iron (Fe), iodine (I), magnesium (Mg), phosphorus (P), potassium (K), sodium (Na) and zinc (Zn).

For the WFR nutrient values were determined with FoodWorks 8 Professional software package (Xyris Software Pty Ltd., Australia). This software linked several national databases available in Australia and allowed new foods to be added (38 generic food items and 19 follow-on and young child formulas were added). For foods specific to Singapore, the Singaporean Health Promotion Board nutrient database [37] was used to create and add new foods into the system (27 items in total). The Composition of Foods Integrated dataset by McCance and Widdowson (revised version) was also consulted [38]. When new foods or recipes were created, and information on all nutrients was not available, efforts were made to match it as closely as possible to an existing food in the database, based on ingredients and nutrient values. The software allowed each nutrient to be over-written with a new value, making it possible to “borrow” the missing nutrient value from a food already in the system. Where brands were given on a WFR, information was obtained from package labelling or company websites. (These were the main sources of information for formulas and supplements.) Breastfeeding was assumed to provide approximately 10 g breastmilk per minute. Per breast, feeds were capped at 10 min, since milk flow after this length of time was considered too slow to contribute nutritionally. Feeds shorter than 2 min were excluded for the same reason. If the next feeding session commenced within 30 min of the start of the previous feed, the duration was added to the first feed and capped at 10 min per breast [39–41].

For the sqFFQ, a reference spreadsheet was developed that included all the nutrient values for the portion specified for each item, using FoodWorks 8. The mean of up to five foods per single item was used to estimate nutrient values. For items which were a group of similar foods, for example, rice-based dishes or small flower fruits, up to five variations of each food in the group were averaged. Each frequency category was converted to a single number of serves per day. For example, 1–3 times a month was averaged to 2 serves/30.4 days = 0.065 serves per day. These were then multiplied by the portion of food to obtain nutrients per day for a particular food. The sum of all the foods in the list was the total intake. As a high proportion of children consumed vitamin and mineral supplements, each type was added to the food list as a new food. Additionally, four new foods were created as they could not fit into an existing category. These were muesli bar, breastmilk, dried seafood, and dried seaweed. Microsoft Office Excel 2010 (USA) was used to determine nutrient intakes per day, which was then exported to a statistical package for analyses.

Nutrients were not adjusted for energy intake. This was deemed unnecessary as the assessment of nutritional intake was not an aim of the present study.

### Statistical analysis

Analyses were performed on IBM SPSS Statistics for Windows, version 23 (IBM Corp., Armonk, N.Y., USA). Anthropometry Z-scores were determined using the World Health Organisation (WHO) AnthroCalc v3.2.2. Data were checked for normality using the Smirnov-Kolmogorov test and visual checks of histograms. As 50% of data were skewed, a number of convenient Box-Cox transformations (cube, square, square root, cube root, natural log, inverse cube root, inverse square root, inverse, inverse square, inverse cube) were performed in an attempt to reduce the skewness to within a range of − 0.5 to 0.5. The cube root values were within this defined range and were used in Bland Altman, correlations and selected multivariable regression models, while raw values were used for other analyses described below. As there was no set method for validating the sqFFQ, a number of techniques documented in the literature were used in a series of steps [42]. A *p*-value of less than 0.05 was considered statistically significant.

#### Correlations between methods

Firstly, linear associations between the two methods were explored using Pearson correlations. Additionally, deattenuated Pearson correlations were used to account for variation in the diet with the formula:

$$ {\mathrm{r}}_{\mathrm{xy}}/\mathrm{sqrt}\left({\mathrm{r}}_{\mathrm{xx}}/{\mathrm{r}}_{\mathrm{yy}}\right) $$

where r_xy_ was the correlation between the mean of the 2-day WFR and first (main) sqFFQ; r_xx_ was the correlation between Day 1 and Day 2 of the WFRs and r_yy_ was the correlation of the first and repeat sqFFQs [43]. Correlations between 0.30–0.49 were considered acceptable and 0.50–0.70 were good [44]. Only nutrients with deattenuated correlations ≥0.30 were included in subsequent analyses.

#### Reproducibility of the sqFFQ

Reproducibility of the sqFFQ was assessed using intra-class correlation (model: two-way mixed; type: absolute agreement; alpha = 0.05).

#### Agreement between methods

The Wilcoxon signed rank test was used to assess differences in median nutrient intakes by each method. Agreement was then assessed using weighted kappa (κ_w_). Quadratic weights were used to assess the statistical significance of the agreement (if sqFFQ and WFR ranked the nutrient into the same quarter = 0 points; adjacent quarter = 1 point, 2-quarter difference = 4 points and extreme quarter = 9 points). This test is generally thought to be a more robust measure than simple percent agreement calculation, since κ_w_ takes into account the possibility of the agreement occurring by chance [45]. κ_w_ was calculated using an online tool developed by Lowry (1998), because SPSS did not have a κ_w_ calculator [46]. Agreements of < 0 indicated poor agreement, 0–0.20 slight, 0.21–0.40 fair, 0.41–0.60 moderate, 0.61–0.80 substantial, and 0.81–1.00 almost perfect [45].

Bland-Altman plots were constructed to assess the differences between the methods for each nutrient. Limits of agreement (LOA) were calculated as:

$$ {\displaystyle \begin{array}{c}\mathrm{Upper}\ \mathrm{limit}=\mathrm{mean}\ \mathrm{of}\ \mathrm{the}\ \mathrm{difference}+\left(1.96\times \mathrm{standard}\ \mathrm{deviation}\right)\\ {}\mathrm{Lower}\ \mathrm{limit}=\mathrm{mean}\ \mathrm{of}\ \mathrm{the}\ \mathrm{difference}-\left(1.96\times \mathrm{standard}\ \mathrm{deviation}\right)\end{array}} $$

(where difference refers to sqFFQ minus WFR for each nutrient), therefore indicating the range in which approximately 95% of data fall. Lastly, using linear regression analyses, the mean was regressed against the difference of the means to check for proportional bias [47].

#### Calibration of the sqFFQ

In the instance where there would be considerable under-, or, overestimation of nutrient intake measured by the sqFFQ, the last step in the validation process was to calibrate the sqFFQ nutrient values against the WFR values, so that it produced similar estimates to the WFR [48]. Multivariable linear regression analyses were used to determine the coefficients needed to derive new calibrated sqFFQ values, using a linear equation (independent variables: original sqFFQ nutrient values, age and/or sex; dependent variable: WFR nutrient values). To ensure the assumptions for multivariable linear regression were met (that is, residuals were normally distributed), certain dependent variable nutrients were entered into the model as cube root values. The final value calculated using the linear equation was back-transformed by cubing. Following this, correlations, comparison of medians, percentile ranks, κ_w_ statistics and Bland Altman plots were repeated with the new data to demonstrate improvement in the performance of the sqFFQ.

## Results

Of the 46 parents who were recruited at the day-care centres, 39 consented and 33 completed the study (completion rate: 12.6%). All 66 parents who were invited via the modified method expressed interest; 65 consented and 62 completed the study (completion rate: 94%). As most of the dropouts occurred towards the end of the study period, they were not replaced. Of the 95 subjects who completed all components of the study, data from 91 participants were included in the following analyses (four subjects had poor quality WFRs). Five of the caregivers who completed the study were fathers and the rest were mothers. The sample was predominantly Chinese, which was reflective of the Singaporean population in 2016 (74.3% Chinese, 13.3% Malay, 9.1% Indian) [32]. The study had slightly more boys than girls, with a median age of 20 months. Table 1 describes other characteristics of the sample.

**Table 1:** Sample characteristics (*n* = 91)

	Median (P25, P75)	*n* (%)
Child		
Age (months)	20.0 (16.0, 28.0)	
Current height (cm)‡	83.0 (78.0, 88.0)	
Current weight (kg)	11.5 (10.0, 13.0)	
Child BMI (kg/m^2^)	16.5 (15.5, 18.2)	
Weight for age *Z*-score (WAZ)	−0.100 (−0.760, 0.770)	
Weight for height *Z*-score (WHZ)^a^	0.49 (−0.24, 1.42)	
Height for age *Z*-score (HAZ)^a^	−0.78 (−1.55, 0.43)	
BMI for age *Z*-score BAZ^a^	0.40 (−0.34, 1.55)	
Child birthweight (g)^b^	3066 (2793, 3444)	
Child birth length (cm)^b^	50.0 (48.0, 52.0)	
Males		50 (54.9)
Females		41 (45.1)
Chinese		63 (69.2)
Malay		16 (17.6)
Indian		12 (13.2)
Parent		
Mother BMI (kg/m^2^)	22.9 (20.2, 25.7)	
Father BMI (kg/m^2^)	25.4 (23.8, 28.1)	
Parental Education Level (father, mother)		
O levels^c^		8 (8.8), 7 (7.7)
A Level/Junior College^d^		3 (3.3), 3 (3.3)
Polytechnic		20 (22.0), 17 (18.7)
Bachelor’s degree		44 (48.4), 52 (57.1)
Postgraduate		15 (16.5), 11 (12.1)
Other		1 (1.1), 1 (1.1)
Total monthly household income in SGD (%)		
< $7000		35 (38.5)
$7000–$15,000		48 (52.7)
> $15,000		8 (8.8)

P25, 25th percentile; P75, 75th percentile; WAZ, weight-for-age z-score; WHZ, weight-for-height z-score; HAZ, height-for-age z-score; BAZ, BMI-for-age z-score

^a^*n* = 89

^b^*n* = 88

^c^General Certificate of Education Ordinary Level; signifies successful completion of secondary school

^d^General Certificate of Education Advanced Level, signifies main school leaving qualification and eligibility to apply for university entry

Pearson correlations between methods were lowest for all fats and vitamins A and E. However, correction for attenuation brought all values up to or above the cut-off of 0.3. The reproducibility of the sqFFQ was high (Table 2). All nutrient values determined by the sqFFQ were significantly higher than the WFR (*p* < 0.001), except for vitamin A, where the difference did not reach significance (Table 3). Table 4 displays the agreement between the methods when intake was ranked into quartiles. κ_w_ values ranged from 0.12 (MUFA) to 0.53 (calcium). Moderate agreement (0.41–0.60) was found for 8 nutrients, energy and 13 nutrients had fair agreement (0.21–0.40), while 4 nutrients had slight agreement (0–0.20).

**Table 2:** Correlations between sqFFQ and WFR (n = 91); correlations for reproducibility of sqFFQ (*n* = 20)

	Before calibration			After calibration		
	Pearson’s r^a^	Deattenuated correlation^a^	Intra-class coefficient (ICC) for sqFFQ1 and sqFFQ2)^a^	Pearson’s r^a^	Deattenuated correlation^ab^	Intra-class coefficient (ICC) for sqFFQ1 and sqFFQ2)^a b^
Total energy	0.39	0.54	0.79	0.57	0.65	0.79
Protein	0.35	0.46	0.87	0.46	0.47	0.42
Total fat	0.28	0.47	0.80	0.42	0.38	0.34
SFA	0.19	0.30	0.84	0.21	–	−0.70
MUFA	0.18	0.30	0.86	0.29	0.43	0.15
PUFA	0.35	0.60	0.91	0.49	0.74	0.35
DHA	0.37	0.54	1.00	0.34	0.40	0.22
Total CHO	0.32	0.44	0.77	0.51	0.58	0.84
Sugars	0.42	0.56	0.97	0.52	0.62	0.42
Fibre	0.56	0.84	0.61	0.57	0.71	0.49
Vitamins						
A	0.26	0.36	0.99	0.40	0.51	0.34
B1	0.50	0.64	0.91	0.48	–	0.38
B2	0.53	0.60	0.97	0.52	0.56	0.76
B3	0.38	0.50	0.87	0.38	0.43	0.09
DFE	0.41	0.56	0.92	0.49	0.54	0.76
B12	0.57	0.67	0.96	0.56	0.63	0.74
C	0.48	0.60	0.96	0.51	0.65	0.42
E	0.25	0.33	0.95	0.23	0.27	0.10
Minerals						
Ca	0.62	0.72	0.97	0.63	0.71	0.59
Fe	0.56	0.67	0.87	0.59	0.62	0.52
I	0.28	0.41	0.92	0.29	0.41	0.16
K	0.47	0.61	0.90	0.51	0.54	0.37
Mg	0.45	0.58	0.83	0.55	0.61	0.46
P	0.45	0.58	0.87	0.55	0.64	0.44
Na	0.47	0.74	0.84	0.47	0.67	0.35
Zn	0.42	0.51	0.96	0.46	0.55	0.35

sqFFQ1, first administration of sqFFQ (also used for validation); sqFFQ2, second administration of sqFFQ; ICC model was two- way mixed model, single measurement type, with absolute agreement definition

^a^Correlations statistically significant (*p* < 0.01)

^b^After calibration, deattenuated correlation could not be computed for SFA and B1; ICC n.s. for total fat, saturated fat, B3 and vitamin E and iodine

**Table 3:** Median, 25th and 75th percentiles for each nutrient measured by the sqFFQ and WFR (n = 91)^a^

	WFR^b^			sqFFQ^b^			Calibrated sqFFQ^c^		
	Median	25th	75th	Median	25th	75th	Median	25th	75th
Total energy (kJ)	4608	3659	5653	6178	5405	8173	4629	3958	5121
protein (g)	40.8	30.4	58.1	57.0	47.0	70.8	43.6	38.8	51.2
total fat (g)	36.7	29.0	43.7	42.8	35.4	55.5	37.2	33.3	40.6
SFA (g)	14.8	11.8	17.7	16.5	12.9	21.1	15.1	14.5	15.9
MUFA (g)	14.1	12.9	17.3	17.1	13.1	21.6	14.5	13.2	15.6
PUFA (g)	6.1	5.0	8.4	8.6	5.9	10.1	6.8	5.9	7.7
DHA (g)	0.10	0.04	0.22	2.80	0.29	4.16	0.12	0.09	0.19
total CHO (g)	143	108	175	214	176	280	145	121	160
sugars (g)	63.7	46.7	85.5	93.8	62.3	130	65.5	56.1	72.9
fibre (g)	8.0	5.8	11.2	14.7	10.9	16.9	7.5	6.5	9.0
Vitamins									
A (μg)	591	413	808	629	484	860	692	600	831
B1 (mg)	1.0	0.6	1.2	1.2	0.8	1.5	0.9	0.7	1.1
B2 (mg)	1.1	0.8	1.5	1.5	1.1	1.8	1.2	1.0	1.3
B3 (mg)	10.0	6.8	12.9	14.1	9.5	17.9	10.2	8.7	11.5
DFE (μg)	260	161	333	374	296	515	259	213	298
B12 (μg)	2.6	1.4	3.4	3.3	2.3	4.0	2.7	2.1	3.1
C (mg)	78.5	40.5	110	113	64.0	145	71.4	59.7	86.4
E (mg)	4.2	2.6	8.5	7.7	5.7	9.5	4.8	4.3	5.2
Minerals									
Ca (mg)	682	455	863	927	689	1312	667	506	795
Fe (mg)	8.0	5.5	10.8	11.6	7.8	15.4	7.6	5.9	9.3
I (μg)	126	104	165	159	125	210	131	123	143
K (mg)	1349	1025	1804	1891	1547	2363	1450	1284	1633
Mg (mg)	150	115	186	219	180	275	142	116	157
P (mg)	747	549	1006	1026	762	1273	692	564	780
Na (mg)	936	480	1235	961	733	1279	777	667	949
Zn (mg)	6.1	4.5	7.5	8.5	6.2	10.9	5.9	5.1	6.6

^a^Differences between medians assessed by the Wilcoxon signed rank test

^b^Before calibration, all median differences except for vitamin A were statistically significant (*p* < 0.001)

^c^WFR and calibrated sqFFQ median was significantly different for vitamin A (*p* = 0.04)

**Table 4:** Weighted kappa (κ_w_) statistics indicating level of agreement between the sqFFQ and WFR (n = 91)

	Before calibration				After calibration			
	κ_w_	Std error	95% CI		κ_w_	Std error	95% CI	
			Lower	Upper			Lower	Upper
Total energy	0.38	0.09	0.21	0.56	0.54	0.09	0.36	0.72
Protein	0.36	0.09	0.19	0.53	0.47	0.08	0.31	0.64
total fat	0.19	0.05	0.09	0.30	0.45	0.09	0.29	0.63
SFA	0.18	0.05	0.08	0.29	0.23	0.09	0.29	0.63
MUFA^a^	0.12	–	–	–	0.23	0.07	0.10	0.36
PUFA	0.41	0.09	0.23	0.58	0.50	0.08	0.34	0.65
DHA	0.40	0.09	0.22	0.58	0.39	0.08	0.22	0.57
Total CHO	0.31	0.08	0.15	0.47	0.41	0.09	0.23	0.59
Sugars	0.46	0.09	0.28	0.64	0.50	0.08	0.34	0.67
Fibre	0.49	0.09	0.31	0.67	0.41	0.09	0.23	0.59
Vitamins								
A	0.16	0.04	0.08	0.23	0.30	0.09	0.14	0.46
B1	0.42	0.10	0.23	0.61	0.41	0.10	0.22	0.60
B2	0.40	0.09	0.22	0.58	0.39	0.09	0.21	0.57
B3	0.29	0.08	0.14	0.44	0.29	0.08	0.14	0.44
DFE	0.40	0.09	0.22	0.57	0.45	0.09	0.27	0.63
B12	0.43	0.09	0.25	0.61	0.44	0.09	0.26	0.62
C	0.40	0.09	0.22	0.58	0.46	0.10	0.29	0.67
E	0.24	0.08	0.09	0.38	0.23	0.07	0.08	0.36
Minerals								
Ca	0.53	0.09	0.34	0.72	0.53	0.09	0.35	0.70
Fe	0.51	0.09	0.33	0.69	0.55	0.08	0.39	0.71
I	0.37	0.09	0.20	0.54	0.22	0.05	0.10	0.27
K	0.34	0.09	0.16	0.52	0.45	0.10	0.26	0.63
Mg	0.21	0.05	0.10	0.32	0.51	0.08	0.36	0.69
P	0.37	0.09	0.20	0.54	0.49	0.09	0.33	0.67
Na	0.43	0.09	0.25	0.61	0.43	0.09	0.24	0.61
Zn	0.36	0.09	0.18	0.53	0.38	0.09	0.18	0.53

^a^standard error and 95% confidence intervals could not be computed for MUFA before calibration

**Fig. 1:**
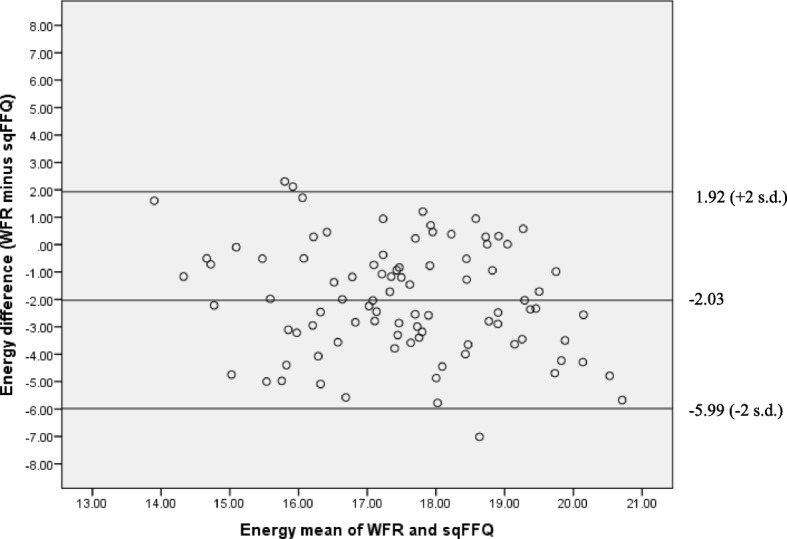
Bland Altman plot for energy before calibration. Indicates mean difference (mid line) and Limits of Agreement (+ 2 standard deviations and − 2 standard deviations); values on the axes are kilojoules transformed to the cube root

Figure 1 illustrates the Bland-Altman plot for energy. The LOAs were wide, indicating large variability in the way the tools measured energy intake. The position of the midline indicated that the sqFFQ overestimated energy intake. This pattern was observed for all nutrients. Linear regression analyses revealed significant proportional bias for energy, SFA, DHA, sugars, fibre and vitamins A, B12 and E. This included both positive and negative trends with increasing intake.

Due to the significant differences observed between the nutrient values obtained from the sqFFQ compared to the WFR, it was necessary to perform the calibration step. Table 5 provides the coefficients used to calibrate the sqFFQ. After calibration, Pearson correlations for energy and 18/25 nutrients improved, and ranged from 0.21 (SFA) to 0.63 (calcium). For the remaining 7 nutrients, correlations remained unchanged or saw slight decreases. After calibration, deattenuated correlations improved for energy and 10/25 nutrients, with no change or a slight decline for the remainder. Deattenuated correlations for two nutrients could not be computed due to negative correlations resulting for the reproducibility of the sqFFQ. One nutrient fell slightly below the range.

**Table 5:** Coefficients for the calibration of the sqFFQ, as determined by multivariable linear regression analysis

	Constant^a^	sqFFQ^b^	Age^c^	Sex^c^	Adjusted R^2^
Total energy (kJ)	2992.629	0.195	58.556	− 675.849	0.306
protein (g)	13.336	0.252	0.739		0.171
total fat (g)	29.570	0.160	0.390	−5.430	0.150
SFA (g)	12.111	0.182			0.043
MUFA (g)^d^	2.508	0.007		−0.137	0.063
PUFA(g)^d^	1.871	0.030		−0.157	0.192
DHA (g)^d^	0.292	0.031	0.006		0.198
total CHO (g)	91.260	0.190	1.930	−26.010	0.260
sugars (g)^d^	3.952	0.005		−0.288	0.225
fibre (g)^d^	1.713	0.033		−0.142	0.313
Vitamins					
A (μg)^d^	10.510	0.001	−0.053	−0.822	0.144
B1 (mg)^d^	0.777	0.158			
B2 (mg)	0.507	0.457			0.264
B3 (mg)	5.599	0.330			0.156
DFE (μg)	181.225	0.223	3.732	−69.446	0.221
B12 (μg)	0.726	0.583			0.268
C (mg)^d^	4.157	0.016		−0.486	0.256
E (mg)^d^	1.464	0.029			0.040
Minerals					
Ca (mg)^d^	7.722	0.002		−0.659	0.388
Fe (mg)^d^	1.783	0.034		−0.150	0.331
I (μg)^d^	4.602	0.003			0.074
K (mg)	394.551	0.326	19.487		0.202
Mg (mg)^d^	4.397	0.003	0.025	−0.333	0.278
P (mg)^d^	7.453	0.001	0.047	−0.536	0.285
Na (mg)^d^	7.270	0.002			0.212
Zn (mg)^d^	1.802	0.024		−0.141	0.193

^a^Constant statistically significant (*p* < 0.05) for all nutrients except protein

^b^sqFFQ statistically significant (*p* < 0.05) for all nutrients except MUFA

^c^Age and/or sex were only included in model if *p* < 0.10; Sex coded 1 for male and 2 for female

^d^The cube root of the WFR value was used for this nutrient. When the coefficients for this nutrient are used in the linear equation to calibrate sqFFQ values, the final value must be cubed to back-transform

Calibration also had varied effects on reproducibility. With the exception of total carbohydrates, reproducibility weakened for other nutrients, and SFA had a negative correlation (Table 2).

Calibration improved the ability of the sqFFQ to rank nutrient intake similarly to the WFR (Table 4). κ_w_ improved for energy and 64% of nutrients; energy and 16 nutrients had moderate agreement (0.41–0.60), and 9 nutrients had fair agreement (0.21–0.40).

**Fig. 2:**
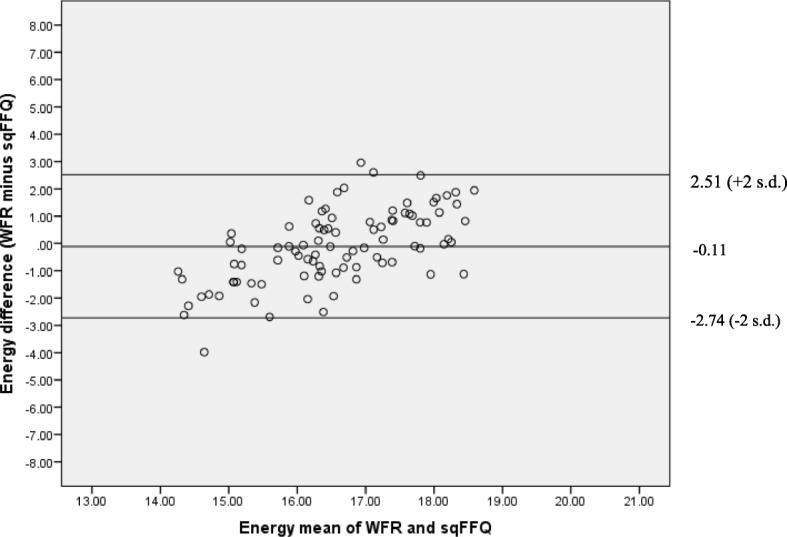
Bland Altman plot for energy after calibration. Indicates mean difference (mid line) and Limits of Agreement (+ 2 standard deviations and − 2 standard deviations); values on the axes are kilojoules transformed to the cube root

Median intakes after calibration for all nutrients were very similar between the methods, with only phosphorus remaining significantly different (Table 3). For all nutrients, Bland-Altman analyses showed mean differences between the methods were now close to zero, with narrower LOAs. Proportional bias was still present for all nutrients, as illustrated visually in Fig. 2. However, overall, the magnitude was reduced and influenced mainly by extremes of intake.

## Discussion

This study aimed to validate a recently developed sqFFQ in its ability to rank and estimate nutrient intakes relative to the WFR, in multi-ethnic Asian toddlers. Results indicated that overall, the sqFFQ overestimated intakes of all nutrients when compared to the WFR. This finding was consistent with literature and most likely attributable to the format of the sqFFQ [26, 27, 48, 49]. With the traditional format of a sqFFQ, not only did parents have to think retrospectively about their child’s habitual intake, but they also had to consider frequency of intake, based on the portion size presented next to each food. For example, if the portion of food that their child consumed was smaller, or, larger than what was specified in the sqFFQ, then the frequency had to also be adjusted accordingly. This procedure had to be repeated for nearly 100 foods, which can be fatiguing for parents (the questionnaire took between 30 and 45 min to complete).

Typically, parents tended to overestimate intake of foods belonging to the breads and cereals, fruit, and meat/poultry/fish food groups. In this sample of children, traditional main meals usually consisted of composite dishes of multiple grains, meat and/or vegetables, and mixed fruit. In the sqFFQ, each type of rice/rice dish has a portion of ½ a bowl, while each meat item had a portion of 1 tablespoon. So, for example, if a child typically consumed ½ a bowl of rice consisting of equal amounts of two grains (brown and white rice) and ½ a tablespoon each of two meat items (pork and fish), 2–3 times a day, parents tended to place a tick in the 2–3 times a day column for each of the four items. This essentially doubled the child’s intake. Ideally, a lower frequency should have been selected to accommodate a smaller portion. This instruction was explained to parents during the initial face-to-face meeting and provided in writing, in the questionnaire. These were the kinds of responses that were flagged for review, and upon further explanation, often parents changed their response to a lower frequency to accommodate the specified portion. However, these types of instructions can be difficult to understand and a flaw of the sqFFQ design. One approach which may reduce this kind of error is to have participants choose a serving size on the FFQ, as well as a frequency, for each item. This would force participants to consider serving size, and it may minimise the need for participants to translate the child’s normal serving size and frequency into the set serving size and corresponding frequency on the FFQ. While answering two questions for each item that the child consumes may seem to increase the workload on the participant, this may in fact be easier than the present requirement.

Another approach would be to make the questionnaire interviewer-administered. It would allow a dialogue between the researcher and participant and the issue could have been addressed immediately, rather than up to two weeks after completion. This approach may not be feasible in large epidemiological studies where thousands of participants are involved. In this instance, a subsample of participants (dependant on study budget) should be interviewed for quality control purposes.

Digitalisation of the sqFFQ may also be an option, so that participants can access the questionnaire via an application on their smartphones or computer [50, 51]. This will provide the participant with a more interesting and interactive experience. For example, Chatbots could be used to clarify participants’ queries, flag unusual responses and prompt the user to think about their selection. Additionally, digital tools could potentially reduce the burden on researchers, if data entry can be replaced with nutrient intakes that are instantly calculated by the application, so that unusual nutrient intakes can be promptly identified and followed up.

Such technology will no doubt have its limits. The data would need to be reviewed for quality, as there is still reliance on the participants’ memory. Unusual responses cannot be eliminated and could potentially increase. How food portions are scaled could also be misleading [51]. The technology may not be accessible in all communities. Lastly, the time and cost invested in these technologies need to be considered as it is better suited to studies with long follow-up and frequent assessment time points.

In these analyses, it was found that total fat, SFA, MUFA, vitamin A and E had the weakest correlations and/or agreements between methods. Again, this was consistent with the literature, particularly for fats [26, 27, 48, 49]. There may be several reasons for this. Firstly, the addition of fats and oils are not typically measured during cooking. Secondly, these items are also not the main ingredient in recipes and could be subconsciously left out in recording. Lastly, such ingredients may not feature prominently in food prepared for this age group, or, food may be cooked in bulk and frozen into small portions making it very difficult to estimate how much was in each portion. Any of these reasons could create a significant discrepancy with the sqFFQ. PUFA and DHA on the other hand, were the only fatty acids to demonstrate much stronger correlations and agreements. It could be that follow-on and young child formulas (formulas for children above the age of one year) were the main contributors of DHA, and intake of this item was captured similarly by both methods. Likewise, calcium had high correlations and agreements, even before any adjustments.

The initial impression of the results indicated that the sqFFQ in its current form may not be suitable for ranking intake of all nutrients, as total fat, SFA, MUFA and vitamin A only had slight agreement. However, it must be noted that the tools are, in fact, measuring different aspects of the diet: habitual versus actual intake. So, some weaker agreements should be expected, relative to the WFR. Perhaps, if the WFR were completed for more days over a longer period of time, it would be more representative of present usual intakes, thus increasing the agreement between the methods. However, this could also reduce the quality of data from FRs as it is difficult to keep accurate food diaries for many days. Overall, the sqFFQ in its current form is suitable for ranking nutrient intakes.

The sqFFQ in its current form should not be used to estimate population intakes, as it would result in substantial overestimation. This finding was consistent with literature, regardless of participant age [16, 48]. If nutrient intakes need to be estimated, then this study demonstrated that the calibration step was effective in: bringing the sqFFQ values closer in range to the WFR values; strengthening agreements for at least two-thirds of nutrients and bringing the mean difference between the tools close to zero for all nutrients. Vitamin A was the only nutrient in the original sqFFQ whose median was not statistically different to the WFR median. Calibration resulted in the median difference becoming significantly different, however, it did improve correlations and agreements for this nutrient. Iodine was negatively impacted as agreement declined, although it remained within the category of “fair” agreement. Lastly, calibration had varied effects on reproducibility. With the exception of total carbohydrates, reproducibility weakened but remained acceptable and SFA had a negative correlation. We speculate weaker and negative correlations were an artefact of the calibration methods aimed at improving agreement between the sqFFQ and WFR. The adjustments via regression equations reduced the range of individual intakes, resulting in poorer apparent reproducibility of the sqFFQ for most nutrients. It must be noted, however, that reproducibility after calibration was only included here for demonstrative purposes. In an actual study, the reproducibility of a new tool only needs to be tested initially. If the calibration step is used to bring values closer to the reference method, repeating ICC is not necessary.

The ability to calibrate the sqFFQ values to bring values closer to the reference method is a particularly important finding for large studies aiming to estimate nutrient intakes. It is well understood that the use of FRs is expensive to implement in studies. Therefore, one option would be to administer the sqFFQ to the whole study group and then select a representative subsample (based on total sample size, age of subjects and study budget), to complete WFRs for internal calibration. This way, nutrient intakes can be estimated for a large sample, without significantly increasing costs and analysis time, compared to having the full sample complete WFRs. Alternatively, if a study had similar participants to the children in this study, then the coefficients provided in Table 5 can be used as a method of external calibration [52].

There are a few limitations to this study. Firstly, the use of non-probability sampling could have resulted in a non-representative sample. However, we were fortunate that the race distribution, parental education level and household income were all reflective of the current Singaporean population [32].

Secondly, weighed food records were kept for two days in this study, which was the minimum number of days reported in validation studies [34]. While two days was sufficient to capture micronutrient intakes in this age group, ideally, up to five days of dietary data would have more accurately accounted for the day-to-day variation in macronutrient intake and the three-month time frame of the sqFFQ [31, 53]. Based on feedback from our pilot study, where participants found keeping the 2-day WFR most challenging, reducing the burden on participants for our main study (to ensure retention and high quality data) was the primary reason for selecting the minimum two days for the reference method.

Presently, there are no recommendations as to whether the full sample was needed to assess reproducibility of the sqFFQ, or, if a proportion of sample was sufficient [34]. Due to the problems faced with recruitment with both the pilot and current study, 20% of the sample was randomly selected to complete the second sqFFQ. This was based on recent studies conducted in children and adolescents, where reproducibility was tested in 10–25% of the sample [27, 36]. While this approach resulted in high correlations (also an effect of the short timeframe between the questionnaires), the findings lacked power. It is therefore recommended that future studies endeavour to have the full sample complete the repeat questionnaire.

Lastly, the sqFFQ asked parents to report on habitual intake over the last three months. In hindsight, this would have been very difficult to estimate; we speculate that some parents may not have even considered this instruction at all. Given how much a toddler’s diet could change over three months, due to both developmental progression and inconsistencies related to illness or experimentation with new foods for example, a two-week retrospective time frame may be more realistic for the parents and produce more accurate results [16].

## Conclusions

To the best of our knowledge, this is the first time that a toddler-specific sqFFQ, developed for a multiethnic Asian population, has been validated against a WFR for an extensive range of nutrients. It is also one of few FFQ validation studies using a range of methods in a systematic way, and therefore provides a model for the conduction of future toddler FFQ validation studies outside of Singapore. This tool will be useful in large epidemiological studies to determine dietary patterns, frequency of consumption of particular foods or food groups, or rank nutrient intakes to study diet-disease relationships. It is recommended that the sqFFQ is interviewer-administered, and only two weeks retrospective to minimise overestimation. While the tool in its current form is not suitable for estimating nutrient intakes of a population, including WFRs in a representative subsample within a study for calibration purposes can overcome this. This allows for more accurate estimation of nutrient intakes in large nutrition studies, without dramatically increasing the time and cost associated with implementing and analysing FRs.

## Abbreviations

FFQ: Food frequency questionnaire
FR: Food record
LOA: Limits of agreement
sqFFQ: Semi-quantitative food frequency questionnaire
WFR: Weighed food record
κ_w_: Weighted kappa
